# *Ricinus communis* L. fruit extract inhibits migration/invasion, induces apoptosis in breast cancer cells and arrests tumor progression *in vivo*

**DOI:** 10.1038/s41598-019-50769-x

**Published:** 2019-10-10

**Authors:** Munmi Majumder, Shibjyoti Debnath, Rahul L. Gajbhiye, Rimpi Saikia, Bhaskarjyoti Gogoi, Suman Kumar Samanta, Deepjyoti K. Das, Kaushik Biswas, Parasuraman Jaisankar, Rupak Mukhopadhyay

**Affiliations:** 1 Cellular, Molecular and Environmental Biotechnology Laboratory, Department of Molecular Biology and Biotechnology, Tezpur University, Tezpur, 784028 Assam India; 2 Division of Molecular Medicine, Bose Institute, P1/12 CIT Road, Scheme VIIM, Kolkata, 700054 India; 3 Laboratory of Catalysis and Chemical Biology, Organic and Medicinal Chemistry Division, CSIR-Indian Institute of Chemical Biology, Jadavpur, Kolkata 700032 India; 4 Institute of Advanced Study in Science and Technology, Vigyan Path, Paschim Boragaon, Guwahati, Assam 781035 India; 5 Present Address: Department of Biotechnology Royal School of Bio-Sciences Royal Global University, Guwahati, Assam 781035 India

**Keywords:** Breast cancer, Cancer prevention

## Abstract

Medicinal plant-based therapies can be important for treatment of cancer owing to high efficiency, low cost and minimal side effects. Here, we report the anti-cancer efficacy of *Ricinus communis* L. fruit extract (RCFE) using estrogen positive MCF-7 and highly aggressive, triple negative MDA-MB-231 breast cancer cells. RCFE induced cytotoxicity in these cells in dose and time-dependent manner. It also demonstrated robust anti-metastatic activity as it significantly inhibited migration, adhesion, invasion and expression of matrix metalloproteinases (MMPs) 2 and 9 in both cell lines. Further, flow cytometry analysis suggested RCFE-mediated induction of apoptosis in these cells. This was supported by attenuation of anti-apoptotic Bcl-2, induction of pro-apoptotic Bax and caspase-7 expressions as well as PARP cleavage upon RCFE treatment. RCFE (0.5 mg/Kg body weight) treatment led to significant reduction in tumor volume in 4T1 syngeneic mouse model. HPLC and ESI-MS analysis of active ethyl acetate fraction of RCFE detected four compounds, Ricinine, p-Coumaric acid, Epigallocatechin and Ricinoleic acid. Individually these compounds showed cytotoxic and migration-inhibitory activities. Overall, this study for the first time demonstrates the anti-cancer efficacy of the fruit extract of common castor plant which can be proposed as a potent candidate for the treatment of breast cancer.

## Introduction

Breast cancer is the leading cause of cancer death among women worldwide^1^. The main treatment regimens for breast cancer like surgery, radiation therapy, chemotherapy, targeted hormone therapy are either expensive or have various side-effects^2,3^. So, studies towards finding efficient and cost-effective therapeutic strategies with minimal side effects are important for expansion of current treatment options for breast cancer patients. Medicinal plants are rich sources of bioactive molecules and can be exploited for application as anti-cancer agents. Alkaloids vincristine and vinblastine from the plant *Vinca rosea* and taxol, paclitaxel derived from the plant *Taxus brevifolia* are well established anticancer agents owing to their microtubule-targeting efficacy^4–6^. Podophyllotoxin, a lignin derived from *Podophyllum peltatum* L. or *P. emodi* and their derivatives are also used as anti-cancer drugs in market^7^. While podophyllotoxin act by inhibiting microtubule assembly, its derivatives like etoposides and teniposides act by interacting with DNA and inhibition of DNA topoisomerase II^8^. Camptothecin, a quinoline alkaloid from *Camptotheca acuminata* also acts as commercial anti-cancer drug, which inhibits the DNA enzyme topoisomerase I^9^. Furthermore, purified plant polyphenols, baicalin and fisetin were also shown to possess anti-cancer and apoptosis inducing activity in breast cancer cell lines^10,11^. Curcumin inhibited NF-kB pathway and subsequently, the expression of inflammatory cytokines CXCL-1 and -2, up regulated during metastasis^12^.

Majority of the breast cancer mortality cases are primarily due to metastasis of the primary cancer to different sites including organs like bones, brain, liver, lymph nodes and lungs^10^. The 5-year survival rate of metastatic breast cancer patients is about 25% suggesting the importance of targeted therapy for metastasis^13,14^. In search of a novel medicinal plant-based therapeutic approach against breast cancer, fruit extract of *Ricinus communis* L. *(Euphorbiaceae)* from North East Indian origin has been studied in detail. North-Eastern part of India is a well-regarded reservoir of traditional medicinal plants as it is one of the prominent biodiversity hotspots of the world^15^. *R. communis* L, commonly known as castor plant, is abundant in North East India and well-known for its traditional and medicinal use globally^16^. In general, various parts of this plant has been used for the treatment of pain, paralysis, constipation, gastritis and warts^17,18^. 50% ethanolic extract of roots of this plants have shown anti-diabetic activity in *in-vivo* rat models^19^. There are other reports which indicate the effectiveness of this plant as anti-fungal agent and also as a pest control measure^19–22^. A volatile extract from the leaves of the plants have shown to induce apoptosis in human melanoma cells (SK-MEL-28)^16^. However, a detailed study on the anti-cancer efficacy of the fruits of *R. communis* L. is not reported.

The current study demonstrates the anti-proliferative activity of *R. communis* L. fruit extract (RCFE) against two breast cancer cell lines MCF-7 and MDA-MB-231. RCFE significantly inhibited migration, adhesion and invasion along with reduction of matrix metalloproteinases 2 and 9 expression. It also induced apoptosis as shown by reduction of anti-apoptotic Bcl-2, induction of pro-apoptotic Bax expression and DNA fragmentation. The induction of apoptosis in both cells was caspase-7 dependent and independent of p53. Interestingly, RCFE inhibited upstream STAT3 activation responsible for induction of MMPs and Bcl-2. RCFE successfully inhibited tumor progression in syngeneic mouse tumor model *in vivo*. HPLC and ESI-MS analysis of the active ethyl acetate fraction of RCFE showed presence of four probable compounds all of which individually showed anti-cancer activities.

## Results

### RCFE induced cytotoxicity in MCF-7 and MDA-MB-231 cells

**Figure 1:**
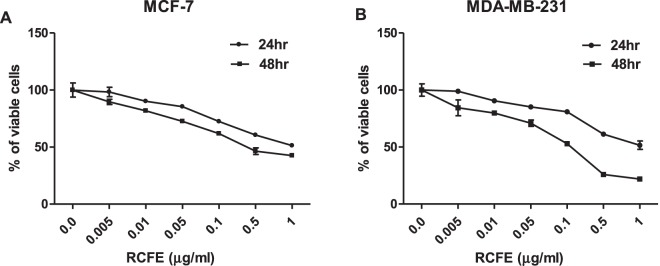
RCFE induced cytotoxicity in breast cancer cells. MCF-7 (**A**) and MDA-MB-231 (**B**) cells were treated with various concentrations of RCFE for 24 and 48 hr. Data represent the mean ± SEM of three independent experiments.

To evaluate the cytotoxic effect of RCFE on breast cancer, MCF-7 and MDA-MB-231 cell lines were treated with various concentrations of RCFE for 24 or 48 hr. RCFE treatment significantly increased cytotoxicity in both the cells in dose and time dependent manner (Fig. 1A,B). Treatment with 1 µg/ml RCFE induced cell death by 48.7% and 55.4% in 24 and 48 hr incubation, respectively in MCF-7 cells (Fig. 1A). Treatment with same concentration led to 48.4% and 78.5% cell death in MDA-MB-231 cells at these time points, respectively (Fig. 1B). To understand the cytotoxic specificity of RCFE, additional cell lines of cancer and normal origin were treated with the extract. Amongst these, HER2-postive MDA-MB-453 and triple-positive ZR-75-1 breast cancer cells showed 36.2% and 54.3% cell death when treated with 1 µg/ml RCFE for 48 hr (Supplementary Fig S1). Similar treatment showed significant cytotoxicity in colon cancer cell line HT-29 (64%) and adenocarcinoma cell line A549 (54.2%) after 48 hr (Supplementary Fig S1). In contrast, treatment with similar doses of RCFE showed minimal effect on the HEK293 and mouse embryonic fibroblast (MEF) cells suggesting the extract’s cytotoxic specificity against cancer cells (Supplementary Fig S1).

### Inhibition of migration, adhesion and invasion of MCF-7 and MDA-MB-231 cells by RCFE

**Figure 2:**
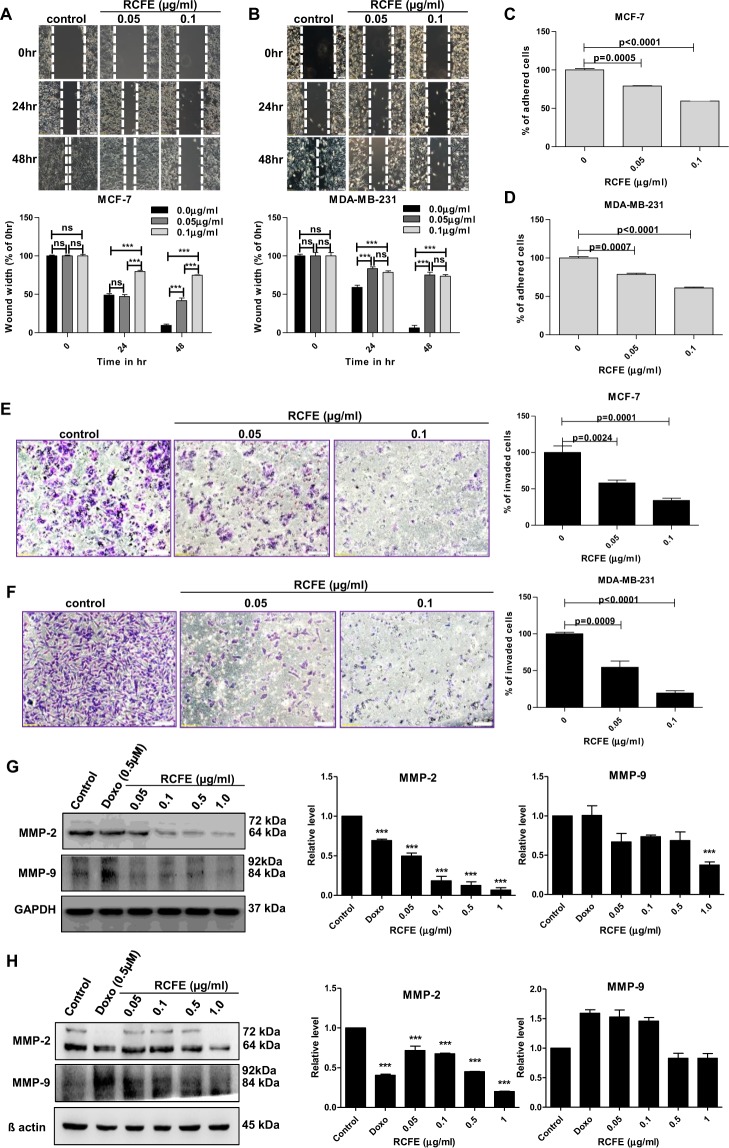
RCFE inhibited metastatic properties of breast cancer cells. Inhibition of migration of MCF-7 (**A**) and MDA-MB-231 (**B**) with treatment of 0.05 and 0.1 µg/ml for 24 and 48 hr. The quantification of wound widths was shown in right panels. Data represent the mean ± SEM of three independent experiments. Statistical differences were analyzed with two-way ANOVA test. p value ns > 0.05, p value *** < 0.0001. Effect of RCFE on adhesion of MCF-7 **(C)** and MDA-MB-231 **(D)**. Data represented as mean ± SEM of three independent experiments. Statistical differences were analyzed with one-way ANOVA test. p value < 0.05 was considered significant. Invasion of MCF-7 **(E)** and MDA-MB-231 **(F)** cells through ECM gel coated transwell inserts in response to RCFE. Data represent the mean ± SEM of five different images of individual set of three independent experiments (shown in right panels). Statistical differences were analyzed with student t-test. p value < 0.05 was considered significant. Western blot analysis of MMP-2 and -9 in MCF-7 **(G)** and MDA-MB-231 **(H)** cells in response to treatment with RCFE. The quantitation of band intensities was represented in bottom panels. Statistical differences were analyzed with one-way ANOVA test. p value < 0.05 was considered significant.

As migration is a primary step in cancer metastasis, we studied the effect of RCFE on migration of MCF-7 and MDA-MB-231 cells using wound healing assay. Near-confluent monolayer of cells was pre-treated with Mitomycin C (1 µg/ml) to confirm the wound healing was due to cell migration and not due to proliferation. RCFE treatment for 24 and 48 hr demonstrated dose-dependent inhibition of migration in both cells (Fig. 2A,B). The inhibition of migration in response to 0.05 µg/ml of RCFE after 24 hr was not significant in MCF-7 cells. However, the same concentration was sufficient to inhibit migration significantly after 48 hr. The effect was highly significant when MCF-7 cells were treated with higher concentration of RCFE (0.1 µg/ml) even at 24 hr. Interestingly, the effect of RCFE on inhibition of migration was robust on highly metastatic MDA-MB-231 as shown by significant inhibition of migration in these cells after treatment with both the doses for 24 and 48 hr (Fig. 2B). Ability to adhere to extracellular matrices is one of the hallmarks of metastatic cancer cells. Pre-treatment of both MCF-7 and MDA-MB-231 cells with two concentrations of RCFE demonstrated significant reduction in adhesion of the cells to collagen IV coated wells in a dose-dependent fashion (Fig. 2C,D). Treatment with 0.05 µg/ml and 0.1 µg/ml RCFE inhibited adherence by 21% and 41% in MCF-7 cells and 22% and 40% in MDA-MB-231 cells, respectively.

Further, the effect of RCFE on invasion of two breast cancer cells was studied. Cells pre-treated with or without RCFE were allowed to invade through extracellular matrix (ECM) gels in response to 10% FBS-containing medium. In MCF-7 cells, treatment with 0.05 and 0.1 µg/ml of RCFE reduced 42% and 66% invasion compared to control cells (Fig. 2E). Interestingly again, the effect was more prominent in MDA-MB-231 cells as treatment with these two concentrations of RCFE led to 45% and 81% reduction in invasion after 6 hr (Fig. 2F).

As these experiments pointed towards anti-metastatic role of RCFE, the expression of metastasis-associated matrix metalloproteinase 2 and 9 (MMP-2 and 9) were studied next. Western blot analysis suggested treatment of RCFE for 24 hr reduced expression of MMP-2 and MMP-9 in MCF-7 and MDA-MB-231 cells in concentration-dependent manner (Fig. 2G,H). RCFE at a concentration of 1.0 µg/ml reduced the MMP-2 expression by about 10 folds in MCF-7 cells and 4 folds in MDA-MB-231 cells. Treatment with the same concentration of RCFE led to ~ 2 folds reduction in expression of MMP-9 in both the cells.

### RCFE induced apoptosis in MCF-7 and MDA-MB-231 cells

**Figure 3:**
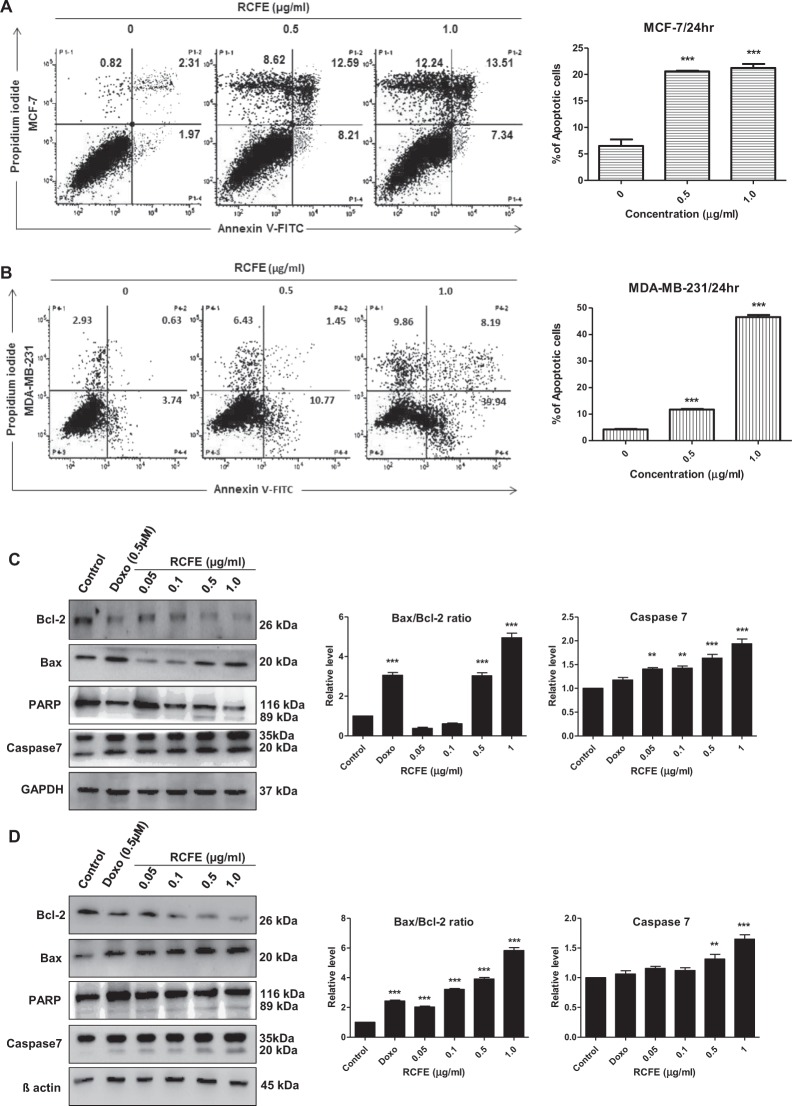
Induction of apoptosis by RCFE. Flow cytometer analysis of MCF-7 (**A**) and MDA-MB-231 (**B**) using Annexin V/PI. The quantitation of three independent analysis was presented in right panels. Western blot analysis of lysates from MCF-7 (**C**) and MDA-MB-231 (**D**) with antibodies against Bcl-2, Bax, PARP and Caspase-7. The ratio of Bax/Bcl-2 and caspase-7 expressions were normalized either to GAPDH or β-actin presented in the right panels. Doxorubicin (Doxo) was used as positive control. Statistical differences were analyzed with one-way ANOVA test. p value < 0.05 was considered significant.

Since, apoptosis is a plausible mode of controlling cancer cell proliferation by an anti-cancer agent; we next studied the role of RCFE in inducing apoptosis in two cell lines. Flow cytometric analysis was performed with cells treated with 0.5 and 1.0 µg/ml RCFE for 24 hr (Fig. 3A,B). In MCF-7 cells, both treatments induced more than 3 folds augmentation in apoptosis (early and late). The increase in apoptosis in MDA-MB-231 cells were found to be 2.7 folds (0.5 µg/ml) and 11 folds (1.0 µg/ml), respectively. Genomic DNA isolated from the cells following treatment with 1.0 µg/ml RCFE for 24 and 48 hr showed degradation of DNA (Supplementary Fig S2).

To understand the signaling mechanism leading to RCFE-induced apoptosis in MCF-7 and MDA-MB-231 cells, expression level of apoptosis regulating proteins like Bax, Bcl-2, PARP, and Caspase-7 were assessed by western blot (Fig. 3C,D). Treatment with RCFE augmented expression of apoptotic protein Bax and concomitantly inhibited anti-apoptotic protein Bcl-2 expression in a concentration-dependent manner in both the experimental cells. This led to increase in Bax/Bcl-2 ratio, critical for cells undergoing apoptosis (Right panels; Fig. 3C,D). Bcl-2 inhibition leads to release of cytochrome c from the mitochondrion which induces the caspase pathway. Treatment with RCFE increased expression level of caspase-7 in dose-dependent manner suggesting onset of apoptosis in the cells (Fig. 3C,D). Significant increase in caspase-7 level was found in cells treated with higher concentrations of RCFE (Right panels; Fig. 3C,D). PARP, a DNA repair enzyme, is a substrate of caspases and increase in PARP cleavage indicates apoptosis in the cells. PARP cleavage as a result of RCFE treatment confirmed caspase-mediated apoptosis in both MCF-7 and MDA-MB-231 cells (Fig. 3C,D). Doxorubicin (0.5 µM) was used as positive control in this study. To understand if cell cycle arrest was also involved in anti-cancer activity of RCFE, expression of Cyclin E1 was studied. In both MCF-7 and MDA-MB-231 cells, the expression of Cyclin E1 did not change significantly with increased concentration of RCFE suggesting cells were not arrested in G1/S phase (Supplementary Fig S3). Expression of tumor suppressor gene p53 was also studied as activation of p53 was proposed to play key role in cell cycle arrest and apoptosis^23^. Treatment with RCFE did not show any activation of p53 in the cells suggesting the RCFE-induced apoptosis in these cells was primarily independent of p53 (Supplementary Fig S3).

### RCFE inhibited phosphorylation of STAT3

**Figure 4:**
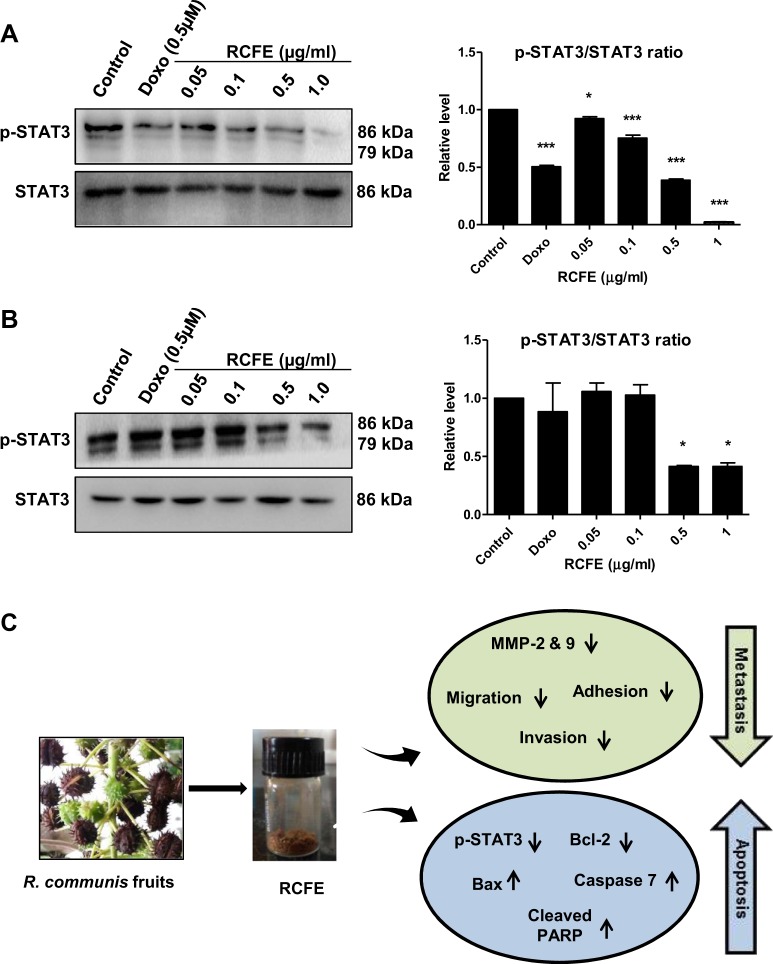
RCFE treatment led to inhibition of STAT3 phosphorylation. Western blot analysis of MCF-7 (**A**) and MDA-MB-231 (**B**) cell lysates with phosphor-STAT3 (Tyr705) and STAT3 antibodies. The quantitation of the expression was presented in right panels. Doxorubicin (Doxo) was used as positive control. Statistical differences of three independent experiments were analyzed with one-way ANOVA test. p value < 0.05 was considered significant. (**C**) Schematic representation of the metastasis inhibition and apoptosis induction by RCFE for its anti-cancer activity.

JAK-STAT pathway is involved in activation of proteins related to apoptosis and metastasis including Bcl-2 and MMP2/9. We investigated the status of STAT3 phosphorylation in these cells followed by RCFE treatment. Treatment with increasing concentration of RCFE significantly reduced phosphorylation at Tyr705 of STAT3, without alteration of total STAT3 protein level (Fig. 4A,B). The reduction in STAT3 phosphorylation was more evident in MCF-7 cells as treatment with 1 µg/ml RCFE almost abolished the phosphorylation. All the above observations pointed towards coordinated role of RCFE in induction of apoptosis and reduction of metastasis in breast cancer cells (represented as a model in Fig. 4C).

### RCFE inhibited tumor growth in syngeneic mouse model

**Figure 5:**
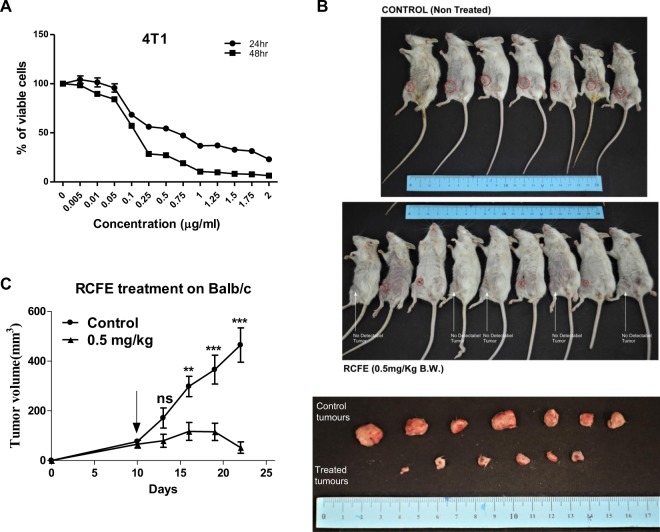
RCFE inhibited tumor progression: (**A**) 4T1 cells were treated with various concentrations of RCFE for 24 and 48 hr. Data represent the mean ± SEM of three independent experiments. (**B**) The animals treated with 0.9% normal saline (upper panel) and RCFE at 0.5 mg/kg bodyweight (middle panel). Animals with visible tumor from outside was pointed with red circles. Animals with no visible tumors from outside was shown by white arrows. The excised tumors from control and RCFE-treated animals (bottom panel). (**C**) Graph represented the measured tumor volumes in two different treatments. Statistical differences were analyzed with two-way ANOVA test. p value ns > 0.05, p value *** < 0.0001.

To understand the effect of RCFE on progression of *in vivo* breast tumor, transplantable mouse mammary carcinoma 4T1 cell induced model was studied. RCFE showed significant cytotoxicity against these cells *in vitro* as shown in Fig. 5A. Tumor was induced in female Balb/c mouse by subcutaneous injection of 4T1 cells in mammary fat pad. After 10 days, intraperitoneal administration of 4 doses of RCFE (at 0.5 mg/kg bodyweight concentration) were given to one set of animals, while the other set of animals received only vehicle (0.9% saline). The tumor continued to increase in the control group while RCFE treated animals showed significant reduction in tumor volume with time (Fig. 5B). The resected tumors from the sacrificed animals at 22 days following 4T1 injection are shown in Fig. 5B (lower panel). RCFE-treated animals showed more than 88% reduction tumor volumes compared to control group animals (Fig. 5C).

### Identification of bioactive components in RCFE

**Figure 6:**
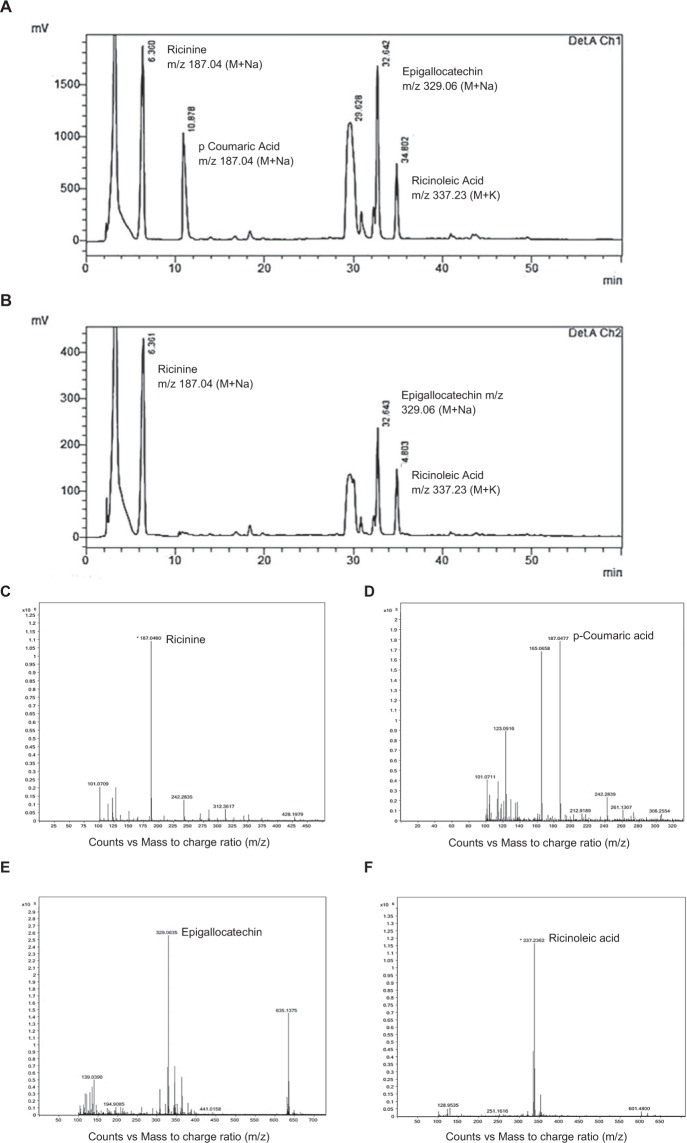
Fingerprint analysis of ethyl-acetate fraction of RCFE. RP-HPLC chromatogram of the ethyl-acetate fraction at a wavelength of (**A**) 210 nm (Det A Ch1) and (**B**) 254 nm (Det B Ch2). ESI-MS spectra of fractions collected after RP-HPLC were identified as Ricinine (**C**), p-Coumaric acid (**D**), Epigallocatechin (**E**) and Ricinoleic acid (**F**).

To identify bioactive components of RCFE, the dried hydro-ethanolic extract was successively fractionated into ethyl acetate, butanol and aqueous fractions. The cytotoxic activity of these fractions was studied which suggested comparatively higher activity in ethyl acetate fraction (Supplementary Fig S4). Hence, this fraction was subjected to HPLC finger printing analysis. Four characteristic peaks were identified in the RP-HPLC analysis (Fig. 6A,B). Retention time, name, molecular formula, molecular weight and chemical structure of the components are shown in Table 1. The major peaks eluted after HPLC was collected, evaporated and their ESI-MS was recorded (Fig. 6C–F). The probable compounds peaks were recognized as Ricinine^24^, p-Coumaric acid^25^, Epigallocatechin^26^ and Ricinoleic acid^25^ by comparing ESI-MS spectra with previously reported literature (Table 1).

**Table 1:** Probable compounds identified by HPLC and ESI-MS technique.

Peak No.	*t*_*R*_ (min)	Comparative Area%	m/z (M^+^)	m/z (M + Na)	m/z (M + K)	Molecular Formula	Compound name & Structure	Reference
**1**	6.36	26.015	164	187.04	—	C_8_H_8_N_2_O_2_		Wachira *et al*.^24^
**2**	10.87	14.387	164	187.04	—	C_9_H_8_O_3_		Wafa *et al*.^25^
**3**	32.64	14.499	306	329.06	—	C_15_H_14_O_7_		Singh *et al*.^26^
**4**	34.80	7.661	298	—	337.23	C_18_H_34_O_3_		Wafa *et al*.^25^

### Cytotoxicity and migration inhibitory activity of pure compounds

**Figure 7:**
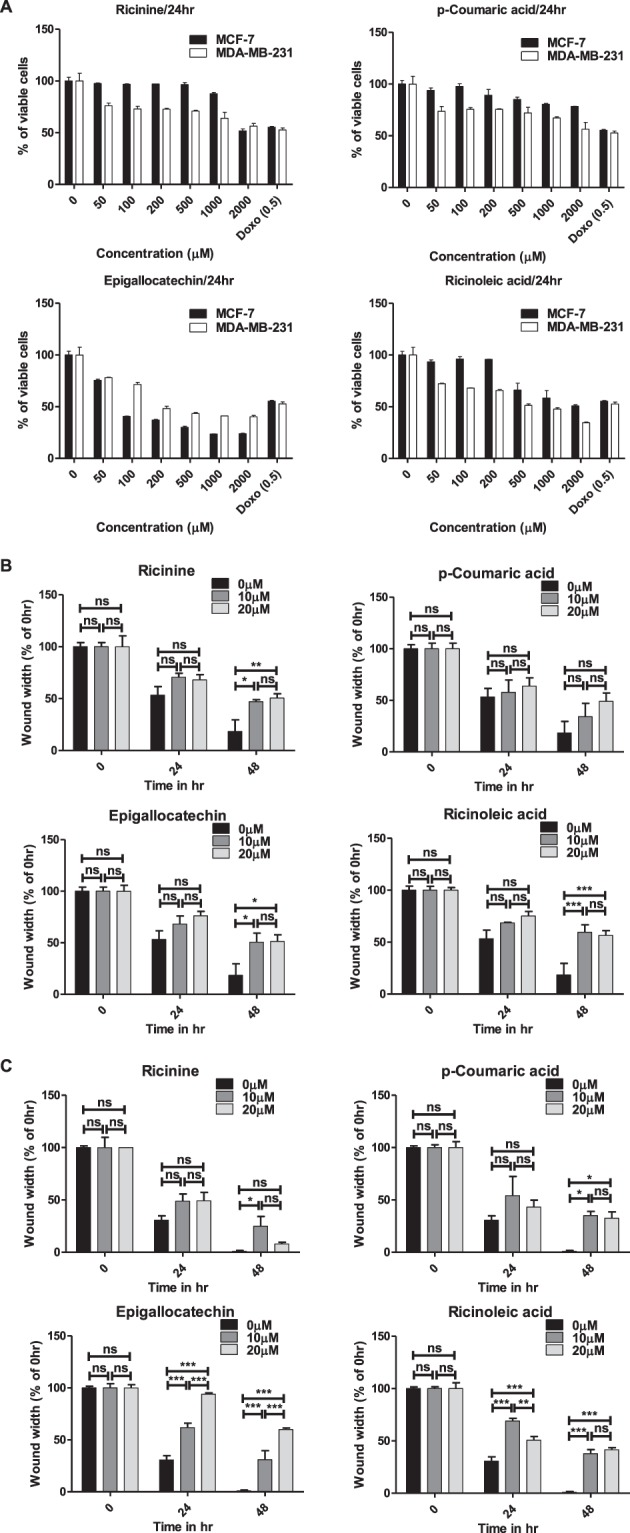
Pure compounds showed significant biological activity. (**A**) The cytotoxic effect of Ricinine, p-Coumaric acid, Epigallocatechin and Ricinoleic acid in MCF-7 and MDA-MB-231 cells after 24 hr treatment. Doxorubicin (Doxo) was used as positive control. Quantitative representation of migration of MCF-7 (**B**) and MDA-MB-231 (**C**) cells by wound healing assay. Data represent the mean ± SEM of three independent experiments. Statistical differences were analyzed with two-way ANOVA test for wound healing assay. p value ns > 0.05, p value *** < 0.0001.

To evaluate the cytotoxicity of four compounds identified from RCFE, MCF-7 and MDA-MB-231 cell lines were treated with increasing concentrations of the Ricinine, p-Courmaric acid, Epigallocatechin and Ricinoleic acid for 24 hr. All four compounds showed cytotoxicity against both cells in a dose dependent manner (Fig. 7A). However, our data suggested that, Ricinine, p-Coumaric acid and Ricinoleic acid were more effective against MDA-MB-231 cells, while Epigallocatechin showed better cytotoxic effect against MCF-7.

Next, the effect of these compounds on migration of MCF-7 and MDA-MB-231 cells was studied using wound healing assay (Supplementary Fig S5). The inhibition of MCF-7 cell migration was not significant for the compounds after 24 hr of treatment with two concentrations of 10 and 20 µM (Fig. 7B). However, the effect was more prominent after 48 hr of treatment which showed inhibition of MCF-7 migration by Ricinine, Epigallocatechin and Ricinoleic acid. The scenario was different in MDA-MB-231 cells where Ricinine and p-Coumaric acid showed no effect after 24 hr treatment and moderate effect after 48 hr (Fig. 7C). Interestingly, Epigallocatechin treatment strongly inhibited MDA-MB-231 cell migration in a dose and time-dependent manner (Fig. 7C). The inhibitory effect of Ricinoleic acid on migration of MDA-MB-231 cells was also highly significant after 48 hr of treatment.

## Discussion

It is now well documented that medicinal plants are a ‘treasure trove’ of bioactive molecules for the treatment of various human diseases. In the last few decades, numerous traditional knowledge-based drugs have been isolated and commercialized^27–29^. Multiple molecules of medicinal plant origin are currently used as drugs to combat cancer (e.g. vincristine, vinblastine, taxol, paclitaxel, Podophyllotoxin). The current study reports the mechanistic details of anti-cancer activities of the fruit extract of *Ricinus communis* L. (RCFE), commonly known as castor bean plant. Oil and seeds of this plant are widely used in folk medicine as purgative, against worm infestation and arthritis. It has also been reported to have anti-inflammatory effects. However, there are no detailed reports on the mechanism of anti-cancer efficacy of this plant.

Metastasis, the property that empowers certain cancer cells to spread into local or distant tissues is a complex process involving migration, adhesion and invasion. These processes can be targeted by an anti-metastatic agent leading to attenuated aggression of cancer cells. Migration of cancer cells to different tissues is an important initial step in metastasis. Treatment with RCFE inhibited migration of both MCF-7 and MDA-MB-231 in a dose-dependent manner. Initiation of metastasis also depends on adhesion property of the cells that involves interaction with extracellular matrix following detachment from the primary sites. RCFE at very low concentrations significantly inhibited adhesion of cells with collagen IV which is an integral part of the basement membrane^30^. The process of invasion is critical for metastasis because the motile cells needs to cross the extracellular matrix and spread into surrounding tissues^31^. In this study, RCFE substantially inhibited efficacy of the cells to invade ECM gel to reach other side of the insert in response to a media containing 10% FBS. The highest inhibition of invasion (81%) was achieved in MDA-MB-231 cells after treatment with a very low concentration of 0.1 µg/ml of RCFE for 24 hr. Remarkably, RCFE showed greater inhibition of cell migration and invasion in highly aggressive triple negative MDA-MB-231 cells compared to MCF-7 cells suggesting its probable application to manage highly aggressive cancer cells. The process of invasion and metastasis is accompanied with degradation of connective tissues and as a result expression of matrix degrading enzymes e.g. matrix metalloproteinases (MMPs) increases. MMP-2 and 9 have been shown to overexpress and contribute to metastatic efficacy of MDA-MB-231^30^. Here, we showed that expression of MMP-2 and 9 were inhibited by RCFE emphasizing its effect on ECM degradation and invasion of cancer cells.

We studied the mechanism of RCFE-induced cell death in MCF-7 and MDA-MB-231 cells. Cell death in these cells via apoptosis was confirmed by flow cytometry analysis using Annexin V/PI. It is clear from the analysis that, RCFE induced apoptosis in a significant percentage of cells. DNA fragmentation assay, a widely used biochemical marker of apoptosis, was performed to confirm this observation. DNA fragmentation occurs at the inter-nucleosomal linker regions in the cells undergoing apoptosis^32–34^. To elucidate the mechanism of apoptosis expression of several pro and anti-apoptotic proteins were studied. RCFE inhibited the expression of anti-apoptotic protein Bcl-2 and induced the expression of pro-apoptotic protein Bax. Expression of Bcl-2 and Bax family proteins plays significant role in the decision of inducing apoptosis in a cell by altering the release of cytochrome c from mitochondria by regulating mitochondrial membrane permeability^35^. Inhibition of Bcl-2 by RCFE possibly induces the cytochrome c production that leads to expression of caspases. Caspases are the key players of apoptosis and a variety of caspases are involved in intrinsic, extrinsic and execution of apoptotic pathway^35^. Caspases use PARP as substrate and cleave PARP during apoptosis^35–37^. We found one of the family members, Caspase 7, a major caspase involved in execution pathway was up regulated in both MCF-7 and MDA-MB-231 validating its role in inducing apoptosis in response to RCFE treatment. Further, increased PARP cleavage with treatment of increasing concentration of RCFE confirmed apoptosis inducing activity of the extract. Expression of p53, a key factor known to induce apoptosis, did not increase suggesting the minimal effect of p53 in inducing apoptosis by treatment of RCFE.

Attenuation of metastasis and induction of apoptosis by RCFE might be attributed to its activity to down regulate phosphorylation of STAT3, a master regulator for these two pathways in cancer cells. Aberrant JAK-STAT signaling (specifically STAT3) is common in various cancer due to their constitutive activation in response to stimulators e.g. cytokines, growth factors, receptors (TLRs/GPCRs), polypeptide ligands and miRNAs^38^. STAT3 induces expression of MMPs and promotes invasion and metastasis in cancer cells. In addition, it induces anti-apoptotic Bcl-2 expression leading to survival of the cells. So, targeting STAT3 has been suggested as a viable therapeutic strategy against cancer^38^. RCFE mediated deactivation of Tyr705 phosphorylation of STAT3 inhibited these two critical steps leading to attenuation of metastatic and induction of apoptosis suggesting possible application of this extract against breast cancer therapy.

The anti-cancer efficacy of RCFE was highlighted by the 4T1 syngeneic mouse model. 4T1 cells are highly tumorigenic and thus suitable for generation of mammary tumors in animals with characters close to human mammary tumors^39^. Our data suggested that 4 doses of intraperitoneal administrations of RCFE at a concentration as low as 0.5 mg/kg bodyweight reduced the tumor volume by about 88% emphasizing its role in limiting breast tumors *in vivo*. Several animal models are reported to study efficacy of drugs against breast cancer: xenograft, genetically engineered (transgenic) and syngeneic models being the most common of them. While xenograft models are popular as it mimics human tumors, it eliminates the possibility of immune response against the tumor leading to host-tumor interaction unnatural to human tumor development^40^. Transgenic animal models overcome this problem and can be used to screen drugs against tumorigenesis. However, genetic changes should be tissue specific in these models, as oncogene-bearing or tumor suppressor gene-knock out systemically may not imitate tumors arising out of mutations in normal microenvironment^40^. These models also take several months to generate tumor and are expensive. Syngeneic models, on contrary, are simple and inexpensive model. Murine adenocarcinoma 4T1 cells implanted in immunocompetent Balb/c, as used in this study, is the most widely used syngeneic model to study tumor progression and metastasis^41^. In future we would like to use this model to understand the efficacy of RCFE to reduce tumor volume through host immune modulation, which would be the subject and focus of an entirely independent study. It would also be interesting to know if the extract would regulate 4T1 cells-induced metastasis in these animals and map the metastatic signaling pathways that might be modulated upon RCFE treatment. We plan to study these aspects in detail to confirm *in vivo* anti-metastatic activity of RCFE.

It is important to have knowledge about constituent molecule(s) of a bioactive plant extract for its probable use as therapy. We performed HPLC and ESI-MS analysis of the ethyl acetate fraction of the extract which revealed the presence of four individual compounds namely, Ricinine, p-Coumaric acid, Epigallocatechin and Ricinoleic Acid. Of them, Ricinine and Ricinoleic Acid have not been reported for any anti-cancer activity albeit their prominent pharmacological importance^42–44^. However, p-Coumaric acid, a hydroxy derivative of cinnamic acid was shown to inhibit proliferation of colon cancer cells in dose-dependent manner^45^. It induced apoptosis accompanied with increasing reactive oxygen species (ROS) levels, a fall in the mitochondrial membrane potential and increased lipid layer breaks. Ethanolic extract of Chinese propolis, where p-Coumaric acid is one of the components, exert antitumor effects mainly through inducing apoptosis of breast cancer cells^46^. Furthermore, Epigallocatechin was also reported for inducing apoptosis and cell cycle arrest^47^. We studied the cytotoxic and migration-inhibitory efficacy of all four compounds to have insight on their individual roles as anti-cancer molecules. Though all four compounds showed cytotoxicity, their efficacy varied with cell types. The inhibition of migration in MCF-7 cells was possibly due to combination of Ricinine, Epigallocatechin and Ricinoleic acid as effect of p-coumaric acid was found to be nominal. Interestingly, in highly metastatic MDA-MB-231 cells, Epigallocatechin contributed most significantly in abrogating migration. However, Ricinoleic acid and Ricinine also contributed moderately to inhibit migration in MDA-MB-231 cells. It may be assumed that the anti-cancer efficacy of RCFE is contributed by synergistic effect of either the identified compounds or their combination with some unidentified compounds.

In summary, our study demonstrated the efficacy of the fruit extract of common castor plant *R. communis* L. against two breast cancer cells of distinctive characteristics. The extract inhibited aggressiveness of the cancer cells by inhibiting characters of metastasis such as cell motility, adhesion, invasion and reduced MMP-2 and 9 expressions. Treatment with the extract induced apoptosis in the cells by augmenting Bax/Bcl-2 ratio that is known to induce caspases and subsequent cleavage of PARP. The phosphorylation of STAT3, a central regulator for activation of metastasis and anti-apoptotic molecules, was inhibited by the extract. The extract significantly reduced tumor volumes in 4T1 syngeneic mouse model. HPLC fingerprinting along with ESI-MS analysis suggested presence of four compounds, all of which showed anti-cancer efficacy individually. The current report contributes significantly to the repertoire of plant-derived therapeutic strategies for the treatment of breast cancer. In future, it would be interesting to study the extract’s role in inhibiting metastasis and modulating immune response for tumor reduction in suitable animal model.

## Methods

### Cell lines and reagents

The cell lines MCF-7, MDA-MB-231, MDA-MB-453, ZR-75-1, HT-29, A549 and HEK293 were purchased from NCCS Pune, India. MEF was a gift from Dr. Sougata Saha, Tezpur University, India. DMEM (Dulbecco’s Modified Eagle Medium) and FBS (Fetal Bovine Serum) were purchased from Life Technologies, USA. MTT (3-(4, 5-dimethylthiazolyl-2)-2, 5-diphenyltetrazolium bromide), Collagen IV, ECM gel and Doxorubicin were also purchased from Sigma Aldrich and Mitomycin C was purchased from HiMedia, India. Annexin-V-FLUOS Staining Kit purchased from Roche, USA and Annexin V FITC Assay Kit was purchased from Cayman, USA. Antibodies used in this study were procured from Cell Signalling Technology, USA.

### Preparation of extracts

Fresh fruits of the plant *Ricinus communis* L. were collected from the Golaghat district of the state of Assam in North East India and morphological identification of the specimen were done at the Botanical Survey of India, Shillong, Meghalaya. The dried fruits were extracted with 50% denatured ethanol at room temperature for 48 hr each time. The extract was filtered and then concentrated under reduced pressure to remove excess ethanol and finally was lyophilised to obtain ethanolic extract (named RCFE). A part of RCFE (5 gm) was then suspended in sterile de-ionised water and partitioned successively with ethyl acetate and n-Butanol. Each fraction was evaporated under vacuum and lyophilized to yield the EtOAc [RC(E)], n-BuOH [RC(B)] and aqueous [RC(A)] fractions. All the fractions were stored at 4 °C and checked for the biological activity.

### Cell culture

MCF-7, MDA-MB-231, MDA-MB-453, HT-29, A549, MEF and HEK293 cell lines were routinely maintained in Dulbecco’s modified Eagle’s medium (DMEM; Gibco), and ZR-75-1 in RPMI1640 supplemented with 10% fetal bovine serum and 1% antibiotic. Cell lines were kept in a CO_2_ incubator at 5% CO_2_ and 37° C temperatures.

### Cytotoxicity assay

In this assay, cells (5000 each) were plated in a 96 well plate and incubated for 48 hr. Cells were treated with different concentrations of RCFE up to 48 hr. Following incubation, cells were treated with MTT and incubated for 3.5 hr. The media was removed carefully and MTT dissolving solution was added and absorbance was taken at 590 nm wavelength using UV-Vis spectrophotometer (Multiscan Go, ThermoScientific).

### Migration assay

Cells were seeded in a 24 well plate till 90% confluency. The media was replaced by FBS-devoid media for at least 6 hr and mitomycin C (1 µg/ml) was added before 1 hr of treatment to stop proliferation. Using a sterile pipette tip, a straight scratch was made simulating a wound in each of the wells. The extract at various concentrations were added and images were taken at 0, 24 and 48 hr following the treatment from at least 3 different fields of each well. The width of the wound was measured and quantified.

### Adhesion assay

To evaluate the efficacy of the extracts to inhibit adhesion, 2 × 10^5^ cells/ml were pre-treated with different doses of extracts in serum free media for 24 hr. Cells were then plated in 96-well plates pre-coated with collagen IV and allowed to adhere for 60 min. The media was gently removed, and the wells were washed. The attached cells were quantified using MTT.

### Invasion assay

2.5 × 10^5^ cells/well were plated in a 6 well plate with or without treatment in a serum free media for 24 hr. Then cells were trypsinised and resuspended in 200 µl serum-free media and placed in the upper chamber of ECM gel pre-coated transwell inserts. The lower chamber was filled with 10% FBS containing media to create a concentration gradient and incubated for 24 hr in case of MCF-7 and 6 hr in case of MDA-MB-231. Then inserts were washed and cells were fixed with formaldehyde and permeabilized with methanol. The cells were then stained with Giemsa stain. Non -invasive cells were removed by scrapping with a cotton swab and bright field images of invasive cells were taken using Olympus IX83 microscope. The cells were counted from photomicrographs of 10 random fields of a single membrane.

### DNA fragmentation assay

Both MCF-7 and MDA-MB-231 cells were treated with RCFE (1 µg/ml) for 0, 24 and 48 hr. Cells were trypsinised and genomic DNA was isolated using PureLink^TM^ Genomic DNA Mini Kit (Invitrogen, USA). Concentration was measured, and DNA was run on 2% agarose gel.

### Flow cytometer analysis

MCF-7 and MDA-MB-231 cells (1 × 10^5^ cells/well) were seeded in 12 well culture plates and incubated for overnight. Then adhered cells were treated with RCFE 0.5 μg/ml and 1 μg/ml for 24 hr. No treatment was given to the control cells. After incubation, both floating as well as adherent cells from each well were collected in tubes and washed with PBS. The cell pellets were resuspended in 200 μL of binding buffer and required proportions of FITC-Annexin V/PI were added to each sample according to manufacturer’s protocol of Annexin V FITC Assay Kit (Cayman, USA). The samples were then allowed to incubate in dark for 15 min and then analyzed with FACSChorus software on a FACS Melody flow cytometer (BD Biosciences).

### Western blot analysis

Cells were seeded in 100 mm dish at 1 × 10^6^ cells per dish and incubated overnight before treating with the indicated concentrations for 24 hr. Proteins were extracted from RCFE treated MCF-7 and MDA-MB-231 cells with ice cold RIPA buffer (Thermo Scientific, USA) containing protease and phosphatase inhibitor cocktail (Thermo Scientific, USA). Equal amount of proteins from different experimental samples was run in SDS-PAGE and proteins were transferred to a PVDF membrane using semidry electrophoresis transfer unit (GE Healthcare, UK). After blocking with 3% BSA in TBS-Tween 20 for at least 1 hr at room temperature, the membranes were probed with the corresponding primary antibody (1:1000 dilutions) overnight at 4° C and secondary antibodies for 1 hr at room temperature. The blots were then incubated with chemiluminescence substrate (Bio-Rad, USA) and bands were visualized using Chemidoc XRS+ system (Bio-Rad, USA). Quantification of the bands was done using Gel Quant software.

### *In vivo* study of mouse tumor model

Female Balb/c mice 6–8-week-old were obtained from Center for Translational Animal Research (CTAR), Bose Institute, Kolkata, India and were maintained as per the guidelines of the animal ethical committee in accordance with the Committee for the Purpose of Control and Supervision of Experiments on Animals (CPCSEA) guidelines. For *in vivo* tumorigenic assay, 4T1 cells (1 × 10^6^ cells/animal) were subcutaneously injected into the mammary fat pad of Balb/c mice to develop solid tumor. Animals with solid tumor were randomly distributed into two groups each containing ten animals. One group was treated with vehicle only (0.9% normal saline) whereas, other group was subjected to intraperitoneal injection of RCFE (0.5 mg/kg) starting after 10 days of tumor development and continued until 22 days (4 doses, 72 hr interval). Tumor progression was monitored by measuring the volume of the tumor with vernier calipers on every third day. The tumor volume was calculated by using the formula V = 0.5 × a × b2, where “a” and “b” indicate length and width diameter, respectively. All animal experiments were conducted in accordance with CPCSEA guidelines and all experimental protocols have been approved by the animal ethics committee of Bose Institute (Ref. No. IAEC/BI/87/2017, dated Dec. 13, 2017) registered with the CPCSEA.

### Fingerprint analysis of *R. Communis L*. fruit extracts

HPLC and ESI-MS techniques was used to identify the phytochemicals present in the *R. Communis* L. extracts. HPLC fingerprint analysis was performed at 25 ± 1 °C using ethyl acetate fraction of *R. Communis* L. which was dissolved in acetonitrile solvent and filtered through membrane filters (0.45 μm pore size). The sample (20 µL injected volume) was analysed using a Shimadzu system (Kyoto, Japan) equipped with LC-20AT Prominence liquid chromatograph pump, DGU-20A_3_ Prominence Degasser, CBM-20A Prominence communications bus module, SPD-20A Prominence UV/VIS detector, LC solution software, and a Rheodyne injector with 100 μL loop.

Separation was achieved using Phenomenex RP C18 column, 250 × 4.6 mm, 5 µm; a gradient mobile phase consisted of water (A) and acetonitrile (B) with a gradient elution program, i.e., 0–40 min, 80–50% B; 40–70 min, 50–0% B; 70–80 min, 0% B; 80–90 min, 0–90% B and 90–100 min 80% B, flow 1 mL/min. The elute was monitored at 210 nm and 254 nm. Mass analysis of the major HPLC peak was recorded on Agilent 6540 Q-TOF LC/MS system.

### Statistical analysis

Statistical analysis was performed using Graph Pad Prism and data were expressed as mean ± standard deviation (mean ± SD). Results were analyzed either by two-way analysis of variance (two-way ANOVA) or one-way analysis of variance (one-way ANOVA) or Student’s t-test as required by the experimental system and difference were considered to be significant at p < 0.05.

## Supplementary information

supplementary information
