# Supplementary material for: Ricinus communis L. fruit extract inhibits migration/invasion, induces apoptosis in breast cancer cells and arrests tumor progression in vivo
Source: Sci Rep. 2019 Oct 10;9:14493. doi: 10.1038/s41598-019-50769-x (PMC6787038; doi:10.1038/s41598-019-50769-x)
Supplement: Supplementary file 1 — supplementary information [file 41598_2019_50769_MOESM1_ESM.pdf]

Supplementary information

Ricinus communis L. fruit extract inhibits migration/invasion, induces apoptosis in breast cancer cells and arrests tumor progression *in vivo*

Munmi Majumder[1], Shibjyoti Debnath[2], Rahul L. Gajbhiye[3], Rimpi Saikia[1], Bhaskarjyoti Gogoi[4], Suman Kumar Samanta[4], Deepjyoti K. Das[1], Kaushik Biswas[2], Parasuraman Jaisankar[3], and Rupak Mukhopadhyay[1]*

[1]Cellular, Molecular and Environmental Biotechnology Laboratory, Department of Molecular Biology and Biotechnology, Tezpur University, Tezpur 784028, Assam, India

[2] Division of Molecular Medicine, Bose Institute, P1/12 CIT Scheme VIIM, Kolkata 700054, India

[3] Laboratory of Catalysis and Chemical Biology, Organic and Medicinal Chemistry Division, CSIR-Indian Institute of Chemical Biology, Jadavpur, Kolkata 700032, India

[4] Institute of Advanced Study in Science and Technology, Vigyan Path, Paschim Boragaon, Guwahati, Assam 781035

***Corresponding author**

Address for communication:

Dr. Rupak Mukhopadhyay

Department of Molecular Biology and Biotechnology

Tezpur University

Assam 784028, India

Email: mrupak@gmail.com; mrupak@tezu.ernet.in

Phone: +91-3712-275417 (Off)

Figure S1:

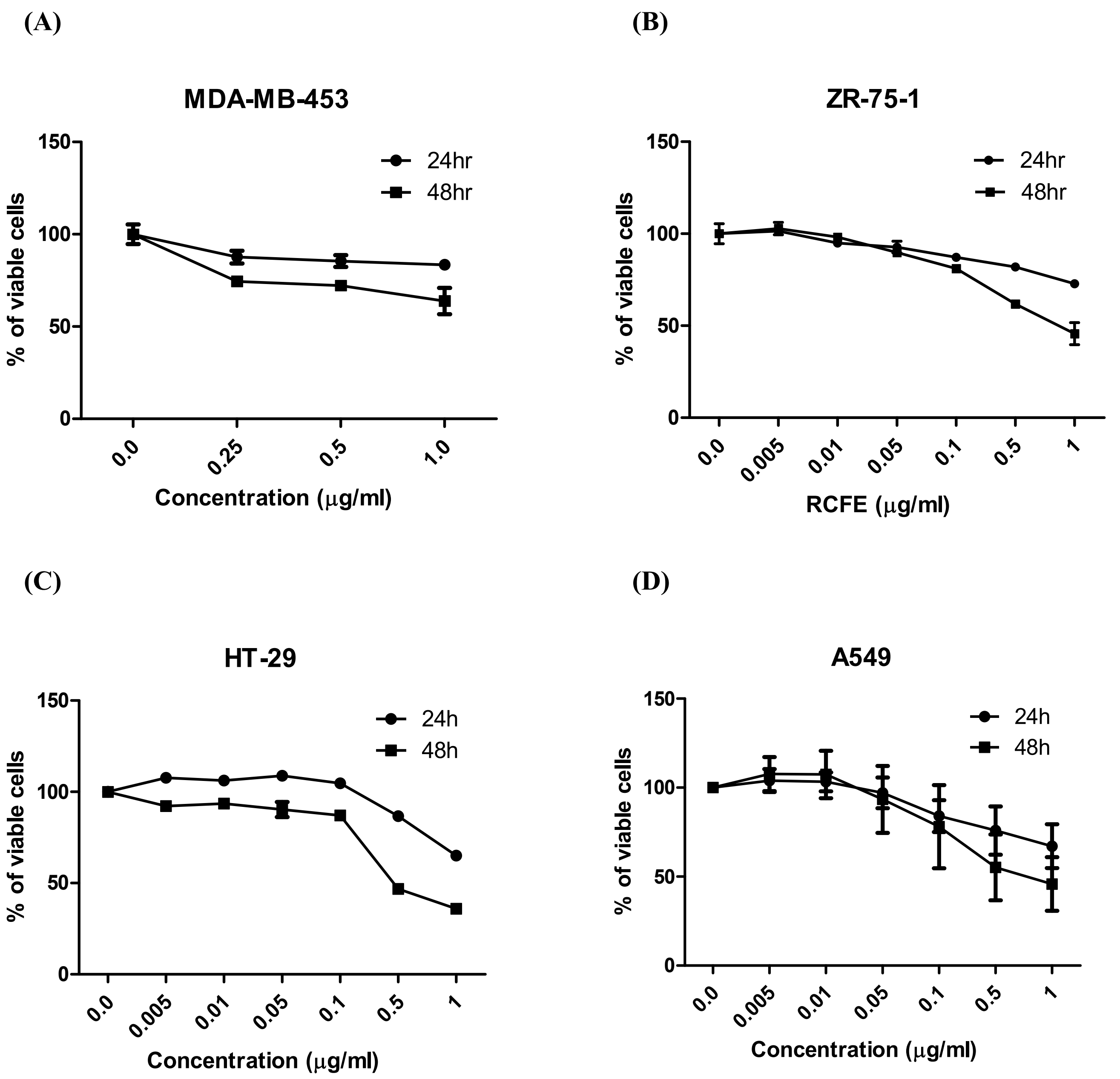
(A)
MDA-MB-453
% of viable cells
150
100
50
0
24hr
48hr
0.0
0.25
0.5
1.0
Concentration (μg/ml)
(B)
ZR-75-1
% of viable cells
150
100
50
0
24hr
48hr
0.0
0.005
0.01
0.05
0.1
0.5
1
RCFE (μg/ml)
(C)
HT-29
% of viable cells
150
100
50
0
24h
48h
0.0
0.005
0.01
0.05
0.1
0.5
1
Concentration (μg/ml)
(D)
A549
% of viable cells
150
100
50
0
24h
48h
0.0
0.005
0.01
0.05
0.1
0.5
1
Concentration (μg/ml)

(E)

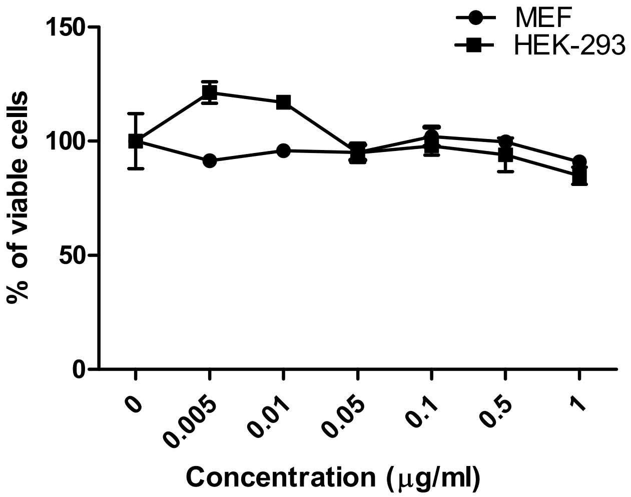

Figure S1. RCFE induced cytotoxicity in several cancer cell lines. Cytotoxicity assay performed with (A) MDA-MB-453 (B) ZR-75-1 (C) HT-29 and (D) A549 cells treated with various concentrations of RCFE for 24 and 48hr. Treatment of HEK-293 and MEF cells with various concentration of RCFE for 24 h (E). Data represent the mean ± SEM of three independent experiments.

Figure S2:

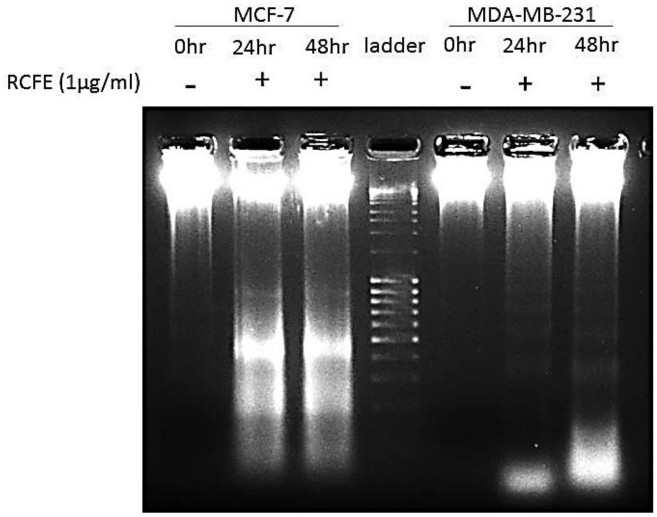

Figure S2. RCFE induced DNA fragmentation in MCF-7 and MDA-MB-231 cells. Genomic DNA isolated from MCF-7 and MDA-MB-231 cells treated with RCFE (1µg/ml) for 24 and 48hr.

Figure S3:

(A)

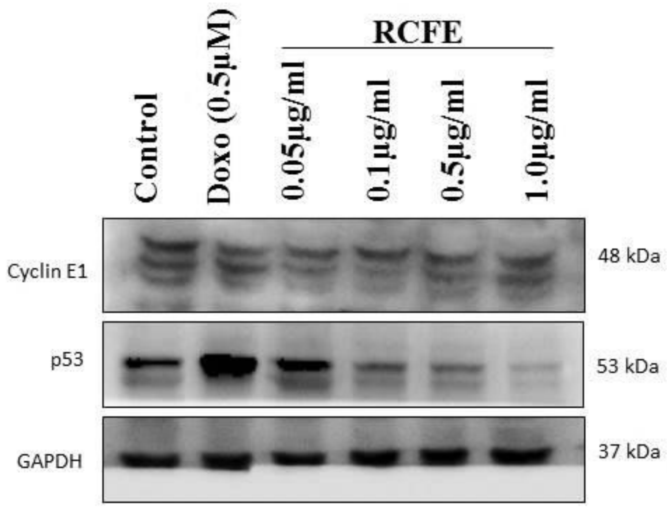

(B)

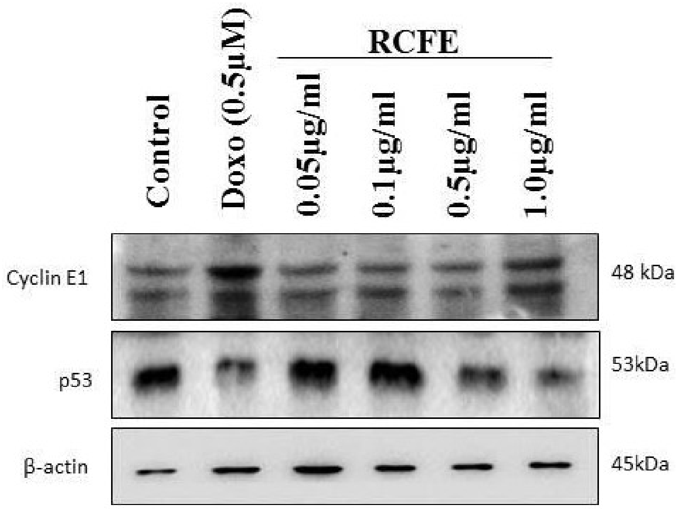

Figure S3. Effect of RCFE on Cyclin E1 and p53 expression. (A) MCF-7 and **(B)** MDA-MB-231 cells were treated with various concentrations of RCFE and expression of Cyclin E1 and p53 were studied using western blots.

Figure S4:

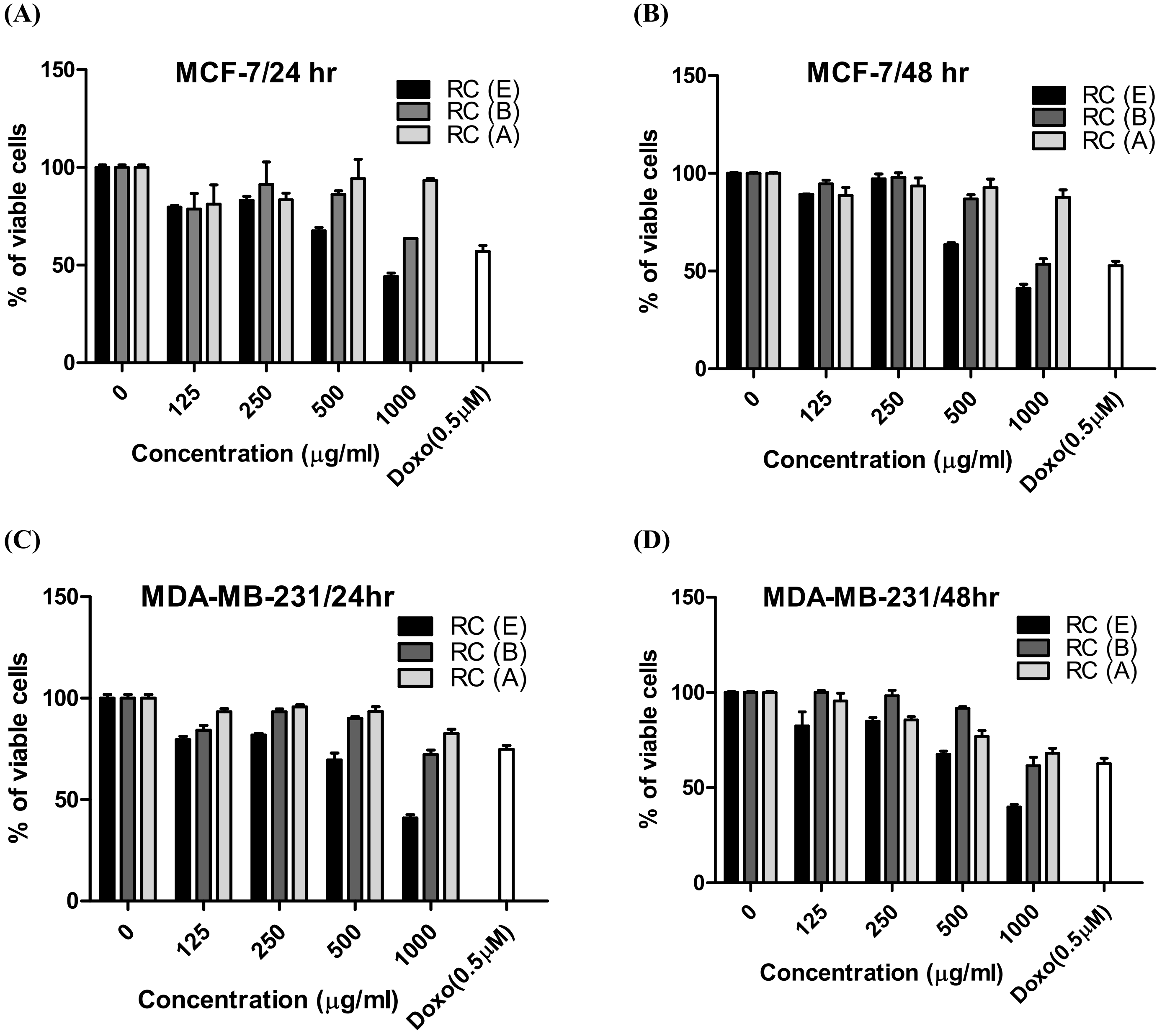

Figure S4. Cytotoxic effect of various fractions of RCFE on human breast cancer cells. Cytotoxic effect on MCF-7 and MDA-MB-231 cells were studied after treatment with three different fractions RC(E) (Ethyl acetate fraction), RC(B) (n-Butanol fraction) and RC(A) (Aqueous fraction) of RCFE for 24 and 48hr. Figure **(A)** and **(B)** represented data of MCF-7 and figure **(C)** and **(D)** represented data of MDA-MB-231 after treatment with each fraction for 24 and 48hr, respectively. Data represent the mean ± SEM of three independent experiments.

Figure S5:

(A)

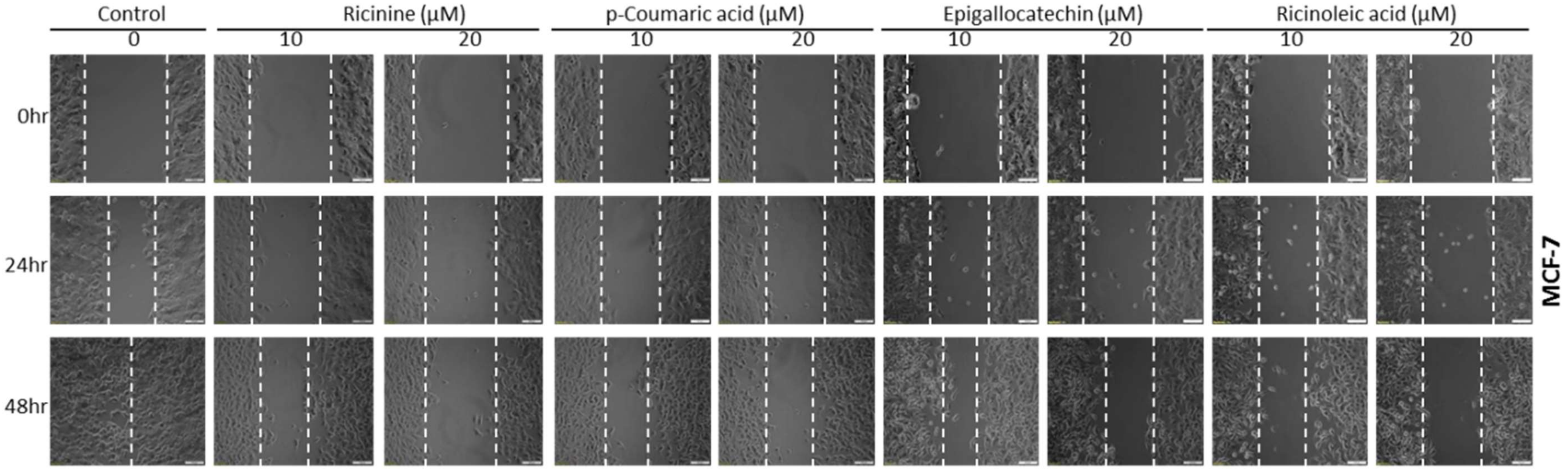

(B)

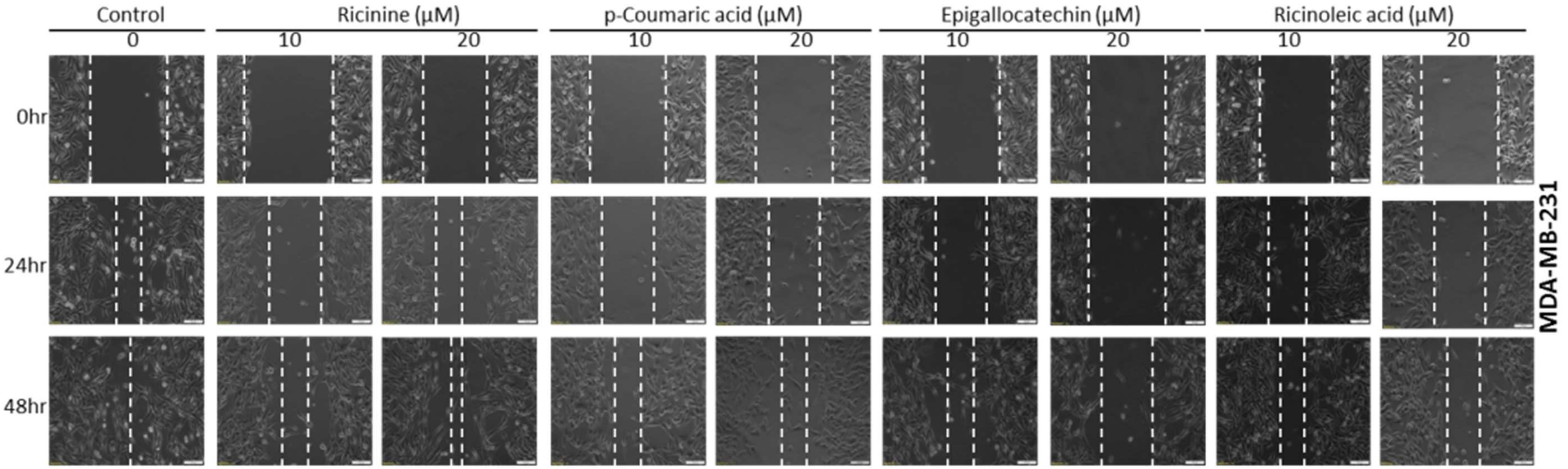

Figure S5. Pure compounds identified from active fraction of RCFE showed inhibition of migration of MCF-7 and MDA-MB-231 cells. Effect of the four compounds Ricinine, p-Coumaric acid, Epigallocatechin and Ricinoleic acid at concentrations of 10 and 20μM on migration of MCF-7 and MDA-MB-231 cells are shown by wound healing assay in MCF-7 **(A)** and MBA-MB-231**(B)**.

Figure S6:

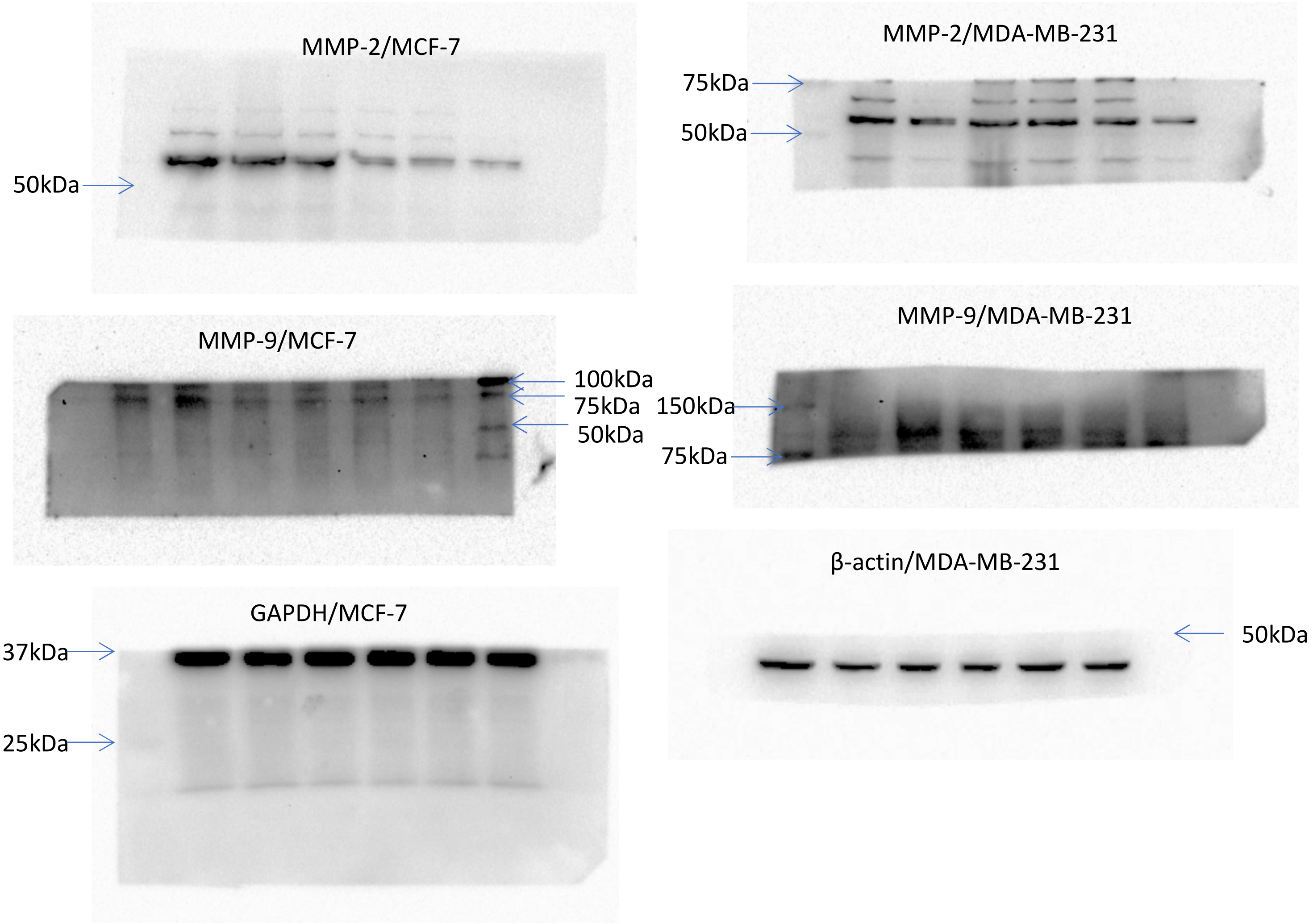

Figure S6: Full-length blots for Figure 2

Figure S7:

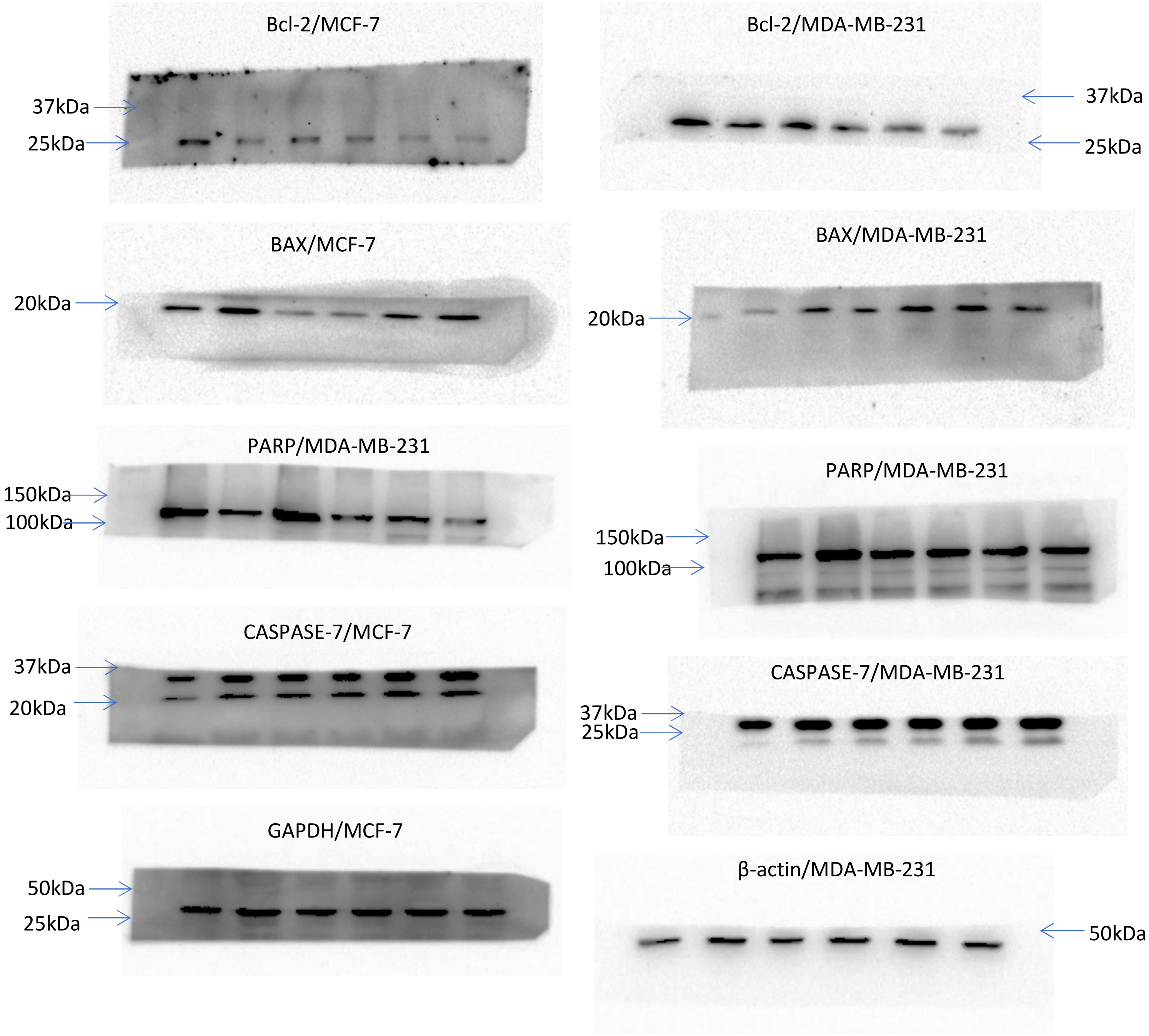

Figure S7: Full-length blots for Figure 3

Figure S8:

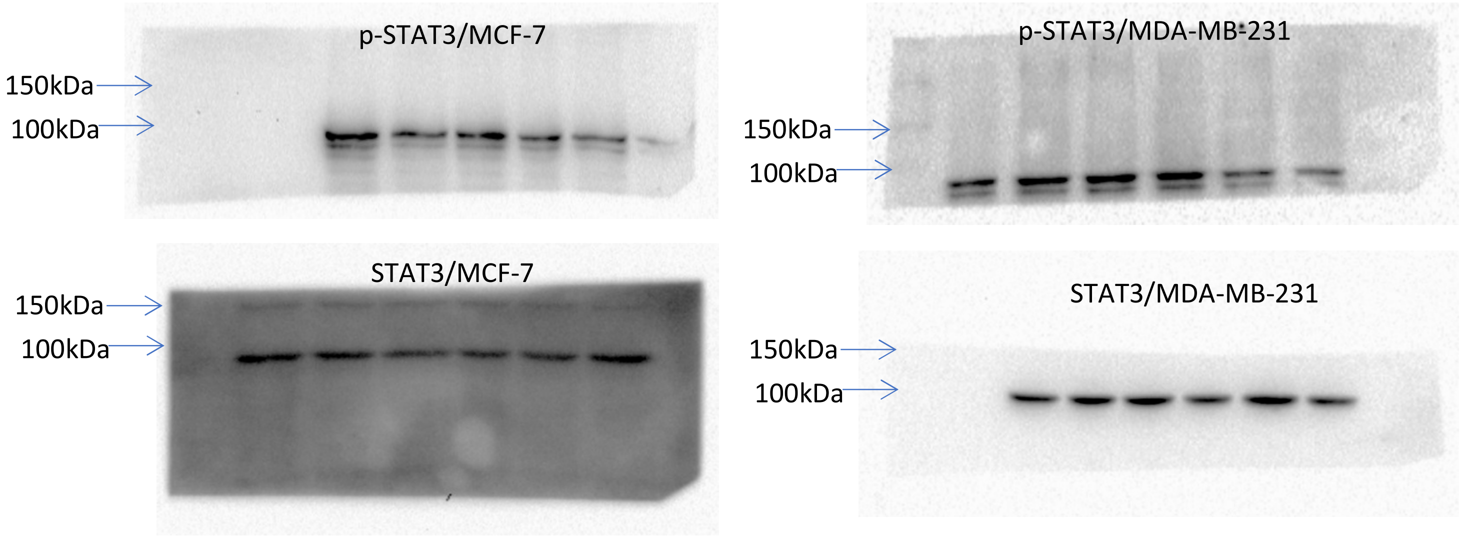

Figure S8: Full-length blots for Figure 4

Figure S9:

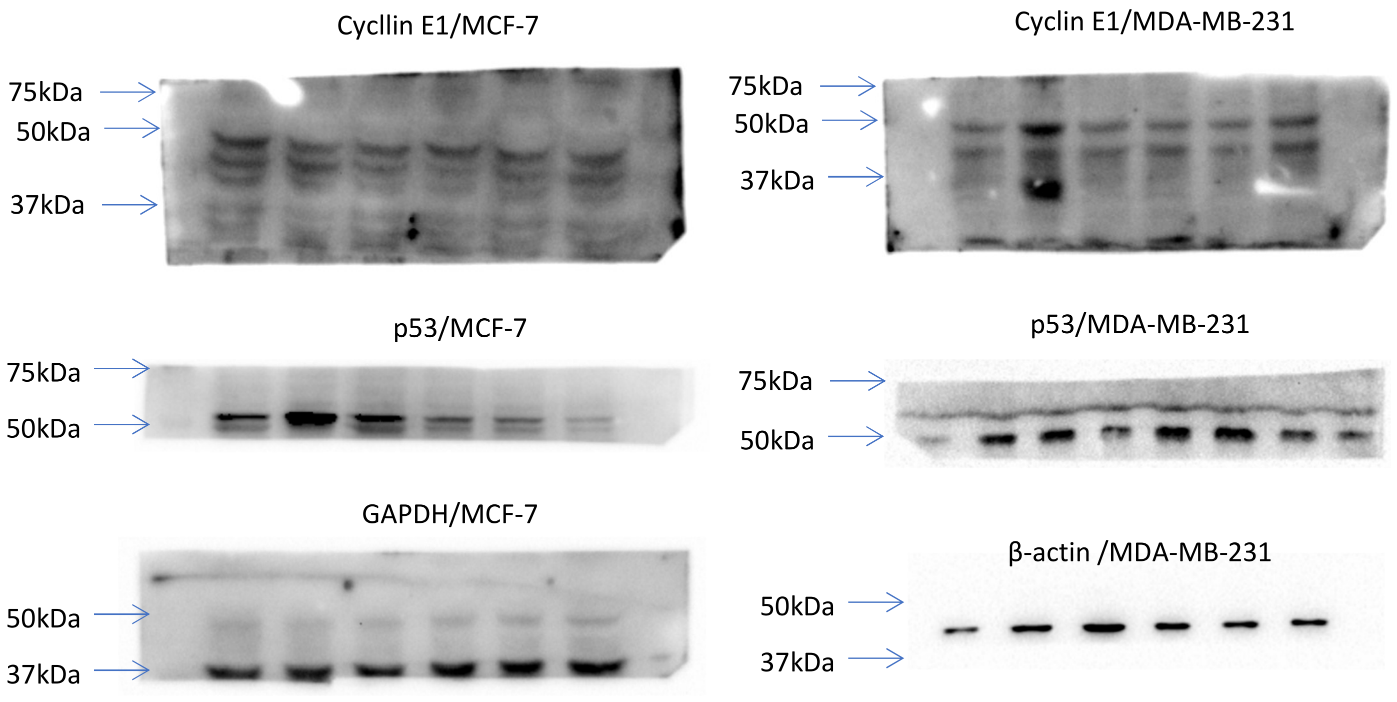

Figure S9: Full-length blots for Figure S3
